# Need for Orthodontic Treatment and Self-Perception in Children Patients of the Faculty of Dentistry of the Nacional del Nordeste University

**DOI:** 10.21142/2523-2754-1304-2025-265

**Published:** 2025-11-08

**Authors:** Paola Berenice Olivera, Edna Yohana Meza, María Natalia Rosende, Alina Noelia Peláez

**Affiliations:** 1 Facultad de Odontología, Universidad Nacional del Nordeste (UNNE). Corrientes, Argentina. pbolivera@odn.unne.edu.ar yohanameza.2@gmail.com mnrosende@odn.unne.edu.ar anpelaez@odn.unne.edu.ar Universidad Nacional del Nordeste Facultad de Odontología Universidad Nacional del Nordeste (UNNE) Corrientes Argentina pbolivera@odn.unne.edu.ar yohanameza.2@gmail.com mnrosende@odn.unne.edu.ar anpelaez@odn.unne.edu.ar

**Keywords:** psychology, maloclussion, mixed dentition, psicología, maloclusión, dentición mixta

## Abstract

**Objective:**

to analyze the need for orthodontic treatment and its relationship with self-perception in children with mixed dentition patients of the Faculty of Dentistry of the National University of the Northeast.

**Materials and methods:**

A cross-sectional observational descriptive study was conducted. For the clinical examination, a fact sheet was presented to the parents and/or guardians of the procedures and objectives of the work signing an informed consent and assent by the patients to be evaluated. The clinical examination was carried out by a single examiner trained for this purpose. All selected patients were given an Orthodontic Treatment Need Index. The analysis of the data was done using the statistical software InfoStat version 2021.

**Results:**

163 patients have been evaluated, 50% correspond to the female gender and 50% to the male sex. Patients were aged between 6 and 11 years, with an average of 7.5 years and a standard deviation of 1.32 years. The Independence test was performed by (χ^2^), we worked with a significance level p = 0.05. between the dental health component of the IOTN and self-perception of the dental aesthetics given by the Aesthetic component of the IOTN, where a correlation between both variables could not be determined.

**Conclusions:**

: no relationship was found between the need for orthodontic treatment and self-perception in children with mixed dentition patients from the Faculty of Dentistry of the UNNE.

## RESUMEN

**Objetivo:**

Analizar la necesidad de tratamiento ortodóncico y su relación con la autopercepción en niños con dentición mixta de la Facultad de Odontología de la Universidad Nacional del Nordeste.

**Materiales y métodos:**

Se realizó un estudio observacional descriptivo transversal. Para el examen clínico, se presentó a los padres o tutores una hoja informativa de los procedimientos y objetivos del trabajo, y se firmó un consentimiento informado y asentimiento por parte de los pacientes a evaluar. El examen clínico fue realizado por un único examinador capacitado para este fin. A todos los pacientes seleccionados se les aplicó un Índice de Necesidad de Tratamiento de Ortodoncia. El análisis de los datos se realizó con el software estadístico InfoStat versión 2021.

**Resultados:**

Se evaluó a 163 pacientes, de los cuales el 50% corresponden al sexo femenino y el 50%, al masculino. Los pacientes tenían entre 6 y 11 años, con una media de 7,5 años y una desviación estándar de 1,32 años. La prueba de independencia fue realizada por (χ^2^) y se trabajó con un nivel de significancia p = 0,05 entre el componente de salud dental de la IOTN y la autopercepción de la estética dental dada por el componente Estética de la IOTN, mas no se pudo determinar una correlación entre ambas variables**.**

**Conclusiones:**

No se encontró relación entre la necesidad de tratamiento ortodóncico y la autopercepción en niños con dentición mixta de pacientes de la Facultad de Odontología de la UNNE.

## INTRODUCTION

Self-perception of the need for orthodontic treatment refers to an individual's subjective sense of necessity for such treatment, encompassing both emotional and cognitive evaluations. Self-perception reflects a person’s ability to understand and assess their own affective, emotional, and mental state. The development of self-image and self-concept begins in childhood within the family environment, influenced by information and experiences from the surrounding environment ^1^.

Numerous studies ^2^^-^^4^ have examined malocclusion severity and the clinical need for orthodontic treatment. However, in recent years, greater attention has been given to studying self-perceived dental aesthetics, a critical factor that often compels individuals to seek orthodontic care ^5^.

According to the World Health Organization (WHO), malocclusions are among the most prevalent oral health issues globally ^6^. These conditions can lead to temporomandibular disorders, dental damage, and issues with speech and swallowing. Beyond their impact on oral health, malocclusions also affect psychological well-being, influencing self-concept and self-esteem ^7^. Therefore, the impact of malocclusions is not limited to functional or aesthetic concerns but also extends to psychological aspects, especially during childhood development ^8^.

As Zielinsky ^9^ suggests, individuals should be viewed as integrated biopsychosocial entities, where biological, psychological, and social factors interconnect. Consequently, a comprehensive approach to treatment is essential rather than a fragmented one. For assessing and documenting occlusal deviations objectively and determining treatment needs in a population, occlusal indices are essential tools, particularly in epidemiological studies ^10^.

Currently, various indices exist to diagnose malocclusions, yet no unified standard defines what constitutes malocclusion or specifies the threshold at which treatment becomes necessary. The interpretation of malocclusion is further influenced by aesthetic preferences, cultural and ethnic factors, and historical trends ^11^.

The Index of Orthodontic Treatment Need (IOTN), developed by Brook and Shaw ^12^, incorporates two components: an objective assessment of dental health and functional indications for orthodontic treatment, as well as a subjective evaluation of the aesthetic impact of malocclusion on the patient’s self-perception.

This study aims to identify the need for orthodontic treatment in children with mixed dentition at an early stage, as this is often the first point of contact between the patient and the dental professional. Early diagnosis is essential to prevent the progression of serious occlusal and psychological issues. Therefore, this research seeks to analyse the need for orthodontic treatment and its relationship with self-perception in children with mixed dentition at the Faculty of Dentistry of the National University of the Northeast.

## MATERIAL AND METHODS

A cross-sectional correlational observational study was conducted. (The study was approved by the Bioethics Committee of the Faculty of Dentistry - Universidad Nacional del Nordeste - ORD Nº 78- 2004- 23/4/2016.) The study population was made up of patients who attended the Clinic of the Comprehensive Care Module for Children and Adolescents of the Faculty of Dentistry of the National University of the Northeast (UNNE), for their comprehensive care in the period from July 2017 to July 2019. For the selection of the sample, patients with mixed dentition between 6 and 12 years old were included, and patients, parents and/ or guardians who agreed to be part of the study with their consent to the use of the exploration data. Patients who presented with any significant systemic pathology with motor or neurological deficiency and those who had received or were receiving at that time, orthopedic or orthodontic treatment were excluded.

 Based on these criteria and according to the formula for the sample calculation, the sample was finally constituted, by 163 patients through a systematic random sampling, to which the Index of Need for Orthodontic Treatment was made.

Clinically evaluated, with the help of a millimeter probe for measurements ^13^, the 5 categories or degrees of treatment need of the Health Component (CS) of the IOTN. The most severe trait identified when examining the patient is what served as a reference to include him within one degree or another, since this index stipulates clear cut-off points between each level.

The categories or grades of the index were evaluated as follows:

Grade 1, which does not need treatment, includes minimal malocclusions with displacements of the contact points less than 1mm. For grade 2, slight need for treatment, highlight greater than 3.5mm but less than or equal to 6mm with competent lips are included; reverse highlight greater than 0mm but less than or equal to 1mm; anterior or posterior crossbite with a discrepancy of 1mm or less between the retruded contact position and the intercuspid position. 

Also, displacement of contact points greater than 1mm but less than or equal to 2mm. Anterior or posterior open bite greater than 1mm but less than or equal to 2mm, increased overbite greater than or equal to 3.5mm without gingival contact and pronormal or posnormal occlusions without other anomalies.

Grade 3, moderate or doubtful need for orthodontic treatment, include in its interval the increased highlight greater than 3.5mm, but less than or equal to 6mm with lip incompetence; reverse highlight greater than 1mm but less than or equal to 3.5mm; anterior or posterior crossbite with a discrepancy of 1 to 2mm between the shunned contact position and the intercuspid position. Displacements of contact points greater than 2mm but less than or equal to 4mm; lateral or anterior open bite greater than 2mm but less than or equal to 4mm; complete deep overbite on the gingival or palatine tissues but without causing trauma.

Severe grade 4 needs treatment, it was considered within this interval to hypodontia or orthodontic closure of spaces before restorative treatment (one tooth per quadrant). Al Resalted increased greater than 6mm but less than or equal to 9m; reverse highlight greater than 3.5mm without difficulty chewing or speaking; reverse highlight greater than 1mm but less than 3.5mm with signs of difficulty chewing or speaking. Anterior or posterior crossbite with more than 2 mm of discrepancy between the returned contact position and the intercuspid position. Posterior lingual crossbite without functional occlusal contact in one or both oral segments. Significant displacements of the contact points, greater than 4mm. Extreme lateral or anterior open bite, more than 4mm. Increased and complete overbite with gingival or palatine trauma. Teeth partially erupted, tilted and impacted against the adjoining teeth. And the presence of supernumerary teeth.

Extreme Grade 5 needs treatment, including impeded eruption of teeth (except third molars) due to crowding, displacement, presence of supernumerary teeth, retained deciduous teeth and any pathological causes. Extensive hypoodontics with restorative repercussions (more than one tooth per quadrant) that needs prepotesic orthodontics. Highlight increased greater than 9mm. Reverse highlight greater than 3.5mm with signs of chewing and speaking problems. Defects of cleft palate and cleft lip and other craniofacial anomalies and submerged deciduous teeth.

In turn, each child evaluated, was made the Aesthetic Component (AC) of the IOTN, the scale of photographs listed on a scale of 1 to 10, and a hand mirror. The patient was asked to first look in a mirror observing his frontal dental appearance, and then choose from among the 10 photographs, which he considered to be more related to his dental aesthetics, without influencing the opinion of the examiner. Only two categories were considered: patients who identified themselves with photos 1 to 7 do not need treatment, and those who were identified with photo 8 to 10 do need treatment.

A descriptive analysis of the data was performed and to evaluate the relationship between the need for treatment and its aesthetic perception.

Statistical analysis: The frequency tables show the absolute and relative frequencies with which the different values of the variables are presented. In order to verify whether the quantitative dental variables presented differences in responses to the aesthetic component, t-tests were performed for two independent samples, with a significance level = 0.05. In order to analyze the frequencies with which the different values of the categorical variables occur and their relationships, concordance tests were performed using the Chi-Square statistic. The data analysis was done using the statistical software InfoStat version 2021.

## RESULTS

163 patients have been evaluated, 50% correspond to the female gender and 50% to the male sex. Patients were aged between 6 and 11 years, with an average of 7.5 years and a standard deviation of 1.32 years. 50% of patients were under 7 years of age, 25% below 6 years of age and 25% below 8 years of age.

**Figure 1:**
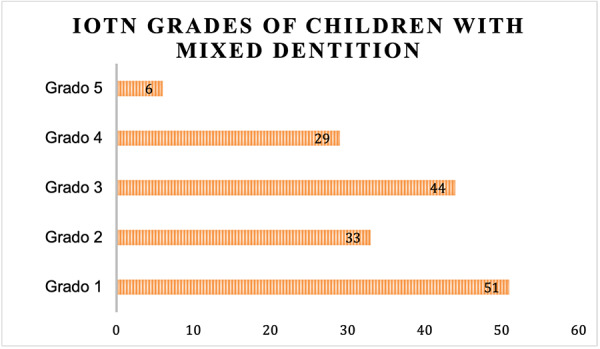
Distribution of the IOTN

The total of the sample was evaluated the Index of Need for Orthodontic Treatment (IOTN) Component of Dental Health, of which it was obtained that 31% (n= 51) of the population, presented Grade 1 that includes a normal occlusion for which it does not need treatment. 27% presented grade 3 (n =44) moderate malocclusions with doubtful need for treatment. Followed by 20% (n= 33) grade 2, with a mild malocclusion that hardly needs orthodontic treatment. In turn, 18% (n =29) of the population presented Grade 4 with severe malocclusions in need of treatment. And only 4% (n= 6) obtained grade 5 with extreme malocclusions and the need for orthodontic treatment (Figure 1)

The same patients were also performed the Aesthetic Component (EC) of the IOTN, of which 63% (n= 102) self-perceived that they do not need orthodontic treatment, while 37% (n =61) perceived that they do need treatment to correct their dental aesthetics.

The Independence test was performed by (χ^2^), we worked with a significance level p = 0.05. between the dental health component of the IOTN and self-perception of the dental aesthetics given by the Aesthetic component of the IOTN, where a correlation between both variables could not be determined.

In order to check whether the quantitative dental variables of the IOTN presented differences between gender and the aesthetic component, t tests were performed for two independent samples, with a level of significance = 0.05. α Table 1 presents the results of the t-tests for independent samples in which the hypothesis of equality between the different perceptions of the aesthetic component for the averages of the quantitative dental variables of the IOTN was tested.

**Table 1:** mean (Me) by aesthetic component (0: no treatment needed, 1: needs treatment), t values for equality of averages and their corresponding probabilities, for the variables: diastema (DIAS), upper jaw highlight (RESMS), lower maxillary highlight (RESMI), overbite (SB), anterior open bite (MAA), posterior open bite (MAP), upper molar irregularity (IRRMS), lower molar irregularity (IRRMI) and posterior cross bite (MCP)

Variable	Medium (0)	Medium (1)	t	p-value	
DAYS	1,18	1,06	0,52	0,601	
Sb	1,06	1,9	2,29	0,024	*
RESMS	2,57	3,34	2,42	0,017	*
RESMI	0,06	0,7	3,48	<0,001	*
Mcp	0,04	0,11	1,50	0,137	
Maa	0,33	0,78	1,42	0,158	
IRRMS	0,45	1,66	3,20	0,002	*
IRRMI	1,15	2,09	1,89	0,061	
MAP	0,09	0,24	1,41	0,162	

(*) indicates significant differences, t- test

For the variable Diastema (DIAS) the t test was performed for the hypothesis of equality between values of the aesthetic component, p-value = 0.6010, indicating that there are no differences in the averages of diastema between patients with different values of the aesthetic component. 

As for the Upper Maxillary Highlight (RESMS): the t-test for the hypothesis of equality between values of the aesthetic component, with a p-value = 0.0177, indicates that the averages of upper maxillary highlight differ between patients who consider that they do not need treatment from those who consider that they need treatment. Patients who consider themselves needing treatment have an average (3.34 mm) higher than those who consider themselves untreated (2.57 mm). On the other hand, the Lower Maxillary Highlight (RESMI): the t-test for the hypothesis of equality between values of the aesthetic component, with a p-value = 0.0009, indicates that the averages of lower maxillary highlight differ between patients who consider that they do not need treatment from those who consider that they need treatment. Patients who consider themselves needing treatment have an average (0.70 mm) higher than those who consider themselves untreated (0.06 mm). 

The Overbite (SB): the t-test for the hypothesis of equality between values of the aesthetic component, a p-value = 0.0243, indicates that the averages of overbite differ between patients who consider that they do not need treatment from those who consider that they need treatment. Patients who consider themselves needing treatment have an average (1.90 mm) higher than those who consider themselves untreated (1.06 mm). 

Anterior open bite (MAA): the t-test for the hypothesis of equality between values of the aesthetic component, with a p-value = 0.1581, indicates that there are no differences in the averages of anterior open bite between patients with different values of the aesthetic component. 

Posterior open bite (MAP): The t-test for the hypothesis of equality between values of the aesthetic component, with a p-value = 0.1626, indicates that there are no differences in the averages of posterior open bite between patients with different values of the aesthetic component. 

The Superior Anterior Molar Irregularity (IRRMS): t-tested for the hypothesis of equality between values of the aesthetic component, with a p-value = 0.0020, indicates that the averages of upper molar anterior irregularity differ between patients who consider that they do not need treatment of those who feel they need treatment. Patients who consider themselves needing treatment have an average (1.66 mm) higher than those who consider themselves untreated (0.45 mm). 

For inferior anterior molar irregularity (IRRMI): the t-test for the hypothesis of equality between values of the aesthetic component, p-value = 0.0611, indicates that there are no differences in the averages of lower molar anterior irregularity between patients with different values of the aesthetic component. 

Posterior crossbite (MCP): The t-test for the hypothesis of equality between values of the aesthetic component, a p-value = 0.1371, indicates that there are no differences in the means of posterior crossbite between patients with different values of the aesthetic component.

## DISCUSSION

This study aimed to analyze the relationship between the need for orthodontic treatment and self-perception in children with mixed dentition using the IOTN as a quantitative tool.

Understanding self-perception of orthodontic treatment need is crucial for effective treatment planning, as criteria for defining attractiveness vary among populations. Many children perceive dental appearance as essential to facial aesthetics, which has been shown to support psychosocial well-being and positively correlate with self-esteem ^15^. Additionally, understanding self-perception in orthodontic treatment provides valuable data for public health initiatives, informing preventive strategies and health promotion efforts that can improve self-esteem and overall stomatognathic health.

Marques et al ^7^ conducted a study to determine orthodontic treatment need using the dental health component (DHC) and aesthetic component (AC) of the Index of Orthodontic Treatment Need (IOTN) among secondary school students. The study found that, according to DHC and AC, 73.33% and 2.4% of the sample required treatment, respectively. Common occlusal traits contributing to higher DHC scores were contact point displacement, hypodontia, and increased overjet. No correlation was found between DHC and AC, aligning with the present study’s findings, where treatment need did not correspond to self-perception.

Guerrero-Luzuriaga et al. ^1^ studied the frequency of self-perceived orthodontic treatment need in 12-year-old schoolchildren in El Sagrario, Cuenca, Ecuador. They found that 18% of the sample perceived a need for treatment, while 82% did not. When categorized by treatment need, 82% showed no need, 13% had moderate need, and only 5% indicated a severe need for self-perceived orthodontic treatment, results that are consistent with those in the present study.

Crespo et al. ^15^ evaluated the impact of malocclusion-related oral conditions on quality of life using the CS Child-IOPD and the IOTN in 11-12-year-old schoolchildren in Azogues, Ecuador. They reported that 31.18% of schoolchildren demonstrated a moderate treatment need-a substantial proportion. However, for the aesthetic component, the majority (91.18%) showed no treatment need. In comparison, the present study found that 63% of children did not perceive a need for orthodontic treatment, indicating slightly lower levels of perceived treatment need.

The findings may be explained by the age range of the participants (6-10 years), as children at this stage are still developing their self-concept and may lack a full understanding of their dental aesthetics. Nonetheless, considering a child’s perception of both dental aesthetics and the need for orthodontic treatment is essential, as it fosters cooperation and motivation, contributing to treatment success.

## CONCLUSION

The study found no significant relationship between the need for orthodontic treatment and self-perception in children with mixed dentition. However, there is a high prevalence of malocclusions in children with mixed dentition, particularly occlusal alterations such as open bite and maxillary irregularities in both the upper and lower jaws. 

From a clinical perspective, involving the child and their caregivers in recognising the importance of orthodontic treatment and its connection to dental aesthetics could improve adherence to the therapeutic plan. Similarly, educating parents about the impact of oral health on their child’s self-esteem and overall development promotes a preventive approach, encouraging care habits that will last throughout their life. 

Addressing orthodontic needs and aesthetic self-perception during the mixed dentition stage is considered not only a way to prevent future complications but also a means to strengthen the physical, emotional, and social development of paediatric patients, making it an essential component of comprehensive dental care.
